# Negative equity – the value of reporting negative results

**DOI:** 10.1242/dmm.050937

**Published:** 2024-08-30

**Authors:** Owen Sansom, Debora Bogani, Linus Reichenbach, Sara Wells

**Affiliations:** ^1^ CRUK Scotland Institute, Glasgow G61 1BD, UK; ^2^ Mary Lyon Centre at MRC Harwell, Harwell Campus, Didcot OX11 0RD, UK

## Abstract

A pervasive discussion point within the scientific community is the value of unpublished or unavailable data. Researchers, funders, ethical review bodies, editors and publishers have all highlighted the need to make more data available to enhance experimental planning and interpretation and to prevent others from repeating similar experiments. This is particularly important in the context of experimentation involving animals and efforts towards replacement, refinement and reduction. However, despite this broad agreement, sharing data that show inconclusive, statistically insignificant or unremarkable results is still not common practice. In this Editorial, we will highlight the value of what are often coined negative (or null) data and outline some emerging initiatives to address the gap between data generated in laboratories and data available to the wider scientific community.

## Teaser

**Summary:** Negative data are often not published or shared, despite their immense value to scientific progression. Here, we outline some initiatives that aim to encourage the dissemination of all scientific results.

## Negative data come in many forms

### Remarkable for being unremarkable – join the Team

Modern biomedical and preclinical research has a formidable task ahead in tackling complex and intractable human health issues, such as cancer and ageing. Collaborations among multiple disciplines have always been the mainstay of progressive research, in which teams with different expertise come together in formal networks to deliver complex projects. This so-called ‘Team Science’ approach allows for the most efficient use of limited resources, such as money, highly qualified or skilled science practitioners, specialised equipment and animals. It also drives better experimental targeting and design, informed by a plurality of minds and datasets, that are more aligned with the challenging multifaceted aspects of disease modelling and investigation.

**Figure:**
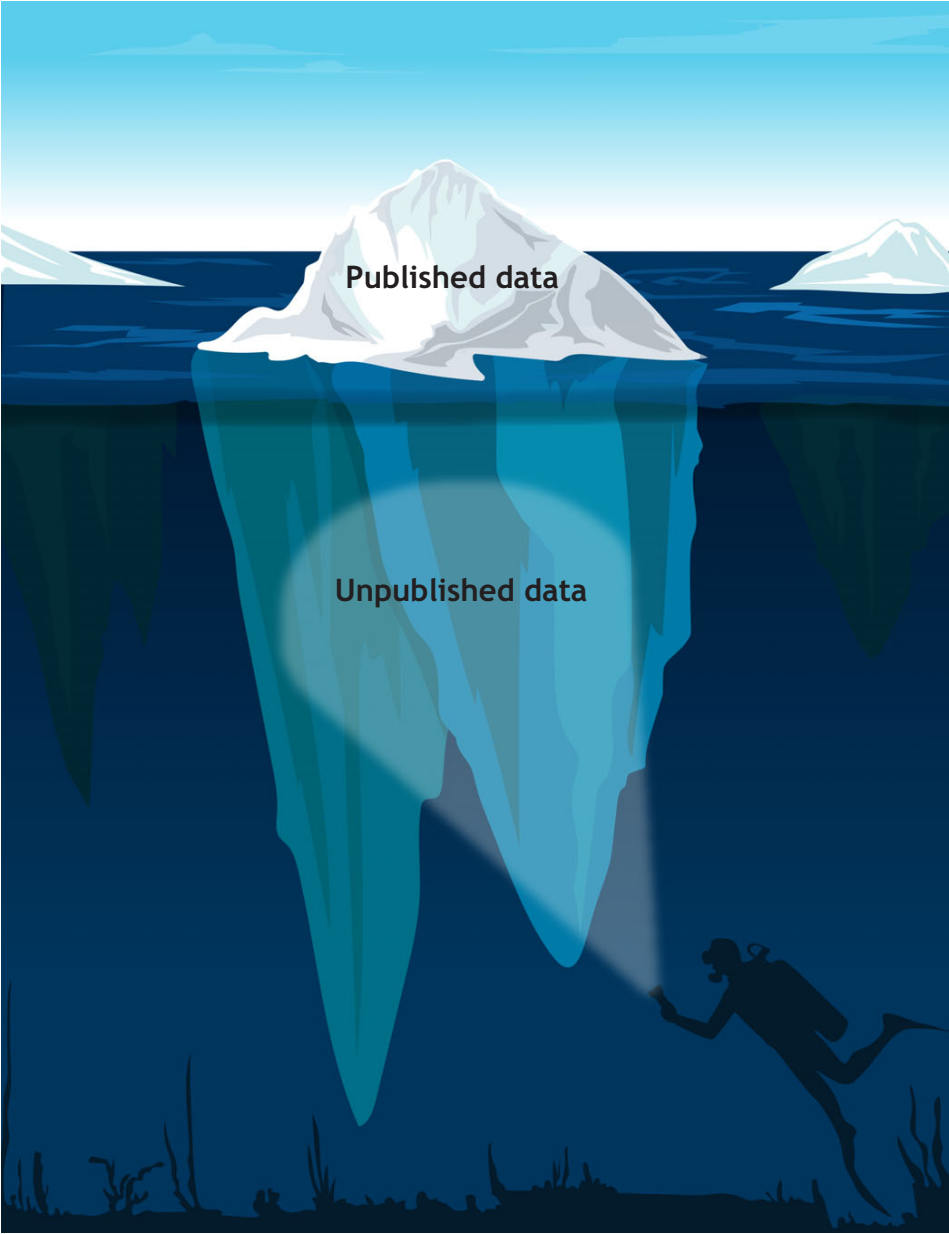


Over recent years, with the availability of extensive data resources, open access publications and extensive web resources, Team Science has become more about global collaborations – not formalised by meetings, grants or contracts, but made possible by free accessibility to data.

The accessibility of multiple datasets derived from diverse experimental designs is enabling a wider perspective on biological and clinical questions. However, there remains a gap between the amount of data generated in laboratories and the amount that is published or available by other means. Data that do not support ground-breaking hypotheses or that are not sufficiently complete to conclude a novel finding often lie dormant in laboratory computers and books. Unfortunately, the lack of peer recognition and publication spaces does not warrant the effort of writing up studies of this nature.

### The reproducibility challenge

In addition to unremarkable data, there are also subsets of data that do not back up already published studies and that are, therefore, difficult to publish or disseminate. This is often because of the acceptance by the research community of the initial findings and a reluctance by many to refute peer-reviewed work. For example, a publication reporting the outcome of a study may describe an initial, positive, theory-confirming result, but multiple subsequent studies are not published as they do not confirm this original result. One such example is a study using transgenic mice in which the amyloid hypothesis was proposed to explain the pathogenesis of Alzheimer's disease. This report was later discredited, but not until it had been widely cited and the study undoubtedly repeated by many researchers who were unable to reproduce the original results (https://www.alzheimers.org.uk/for-researchers/explaining-amyloid-research-study-controversy; Piller, 2022).

Many initiatives have focused on the difficulties of reproducing the results of preclinical studies. If we take preclinical modelling in mice as an example, numerous variables can underlie the differences that are observed between studies, from the genetic background of the mice and variations in diets and equipment, to variables that the research community is only just starting to characterise, such as the microbiome (Ericsson and Franklin, 2021). Deviations from standardised conditions and well-studied variables can reduce generalisation and reproducibility but can also be a powerful tool for discovery (Voelkl et al., 2021). For example, one excellent recent study that examined colitis in a well-established mouse model of this disease identified specific bacterial species that exerted a dominant effect on disease induction and outcome (Forster et al., 2022). Remarkably, colonisation with bacteria was variable within a single animal facility, suggesting that such variation is not simply an inter-facility effect. Interestingly, sequencing for the presence of these bacteria has not been adopted as standard practice for the publication of colitis studies in mice, and other variables are similarly overlooked in preclinical studies.

### The drugs don't work… or do they?

Therapeutic intervention studies in model organisms are also heavily under-reported. Drug treatments of animals that result in no amelioration of a disease phenotype (Hayes and Hunter, 2012) or, even more potentially impactful, that lead to catastrophic side effects are often left unpublished. Rarely do researchers report the unsuccessful response of an experimental model to a drug that they had predicted would have an effect, even though the possibility of this drug being retested by others is likely.

Many published *in vivo* studies using therapeutics also omit supporting pharmacokinetic and biomarker data that are critical to understand the concentration of the therapeutic delivered in the experiment, as well as evidence of the degree to which the target has been inhibited. This information is essential for the correct interpretation of each experiment. Moreover, for small-molecule therapeutics, there can be issues with off-target effects at higher doses. A clear understanding of how selective a compound is at a given dose in the experimental system is essential. For therapeutics that are being used in patients, the preclinical doses should reflect the clinical concentrations or pharmacodynamic effects achieved during the trials. Without complete datasets, it can be difficult or near impossible to interpret the results of such studies, regardless of whether there is a positive or negative outcome. In addition, reporting of body weight and additional associated toxicities can be of help in interpreting the value of a specific *in vivo* experiment. One relevant example in the cancer research field is provided by data obtained on inhibition of the hedgehog signalling pathway by cyclopamine (Incardona et al., 1998). Initially, this hedgehog inhibitor was shown to shrink tumours in a mouse model of intestinal neoplasia (C57BL/6J-*Apc^Min^*/J) (Moser et al., 1990). However, mice treated with the reported dose of cyclopamine failed to thrive and hence developed fewer or smaller tumours, and preclinical trials with more selective inhibitors that did not induce toxicity could not reproduce the initial findings. For an excellent review on this particular body of work, see Thazhackavayal Baby et al. (2024).

One key question often asked of a preclinical disease model is how predictive it can be of the human phenotype. Given the attrition of clinical trials moving from phase 1 to phase 3 and the high cost associated with clinical trials, data pointing to low or null efficacy of a compound in a complex disease model could save significant human and financial resources. In the cancer field, although multiple potential factors could explain why compounds might work in a model but not in a patient tumour (e.g. lack of heterogeneity, environmental factors, differences in the stroma and immune cell population, etc.), attention should be paid to data that indicate that treatments may not be successful in preclinical models. It is interesting to note that in colorectal cancer, studies using relatively simple genetically engineered mouse models predicted that MAPK and mTOR inhibition was unlikely to be effective in patients and this was confirmed in costly phase 3 trials. The availability of all relevant data, negative or positive, would allow a better-informed decision on the progression of clinical trials and result in financial resources being better targeted towards therapies with higher chances of success.

## What should be best practice?

There are some very good examples of best practice. Over the last decade or so there have been multiple efforts across model species to bring together phenotyping data. One such effort is that of the International Mouse Phenotyping Consortium (https://www.mousephenotype.org/), which has been phenotyping thousands of mice carrying null alleles through standardised pipelines and publishing data both demonstrating phenotypes and reporting instances in which the gene change did not result in any phenotyping change.

In drug discovery and development, data generated in non-clinical models, such as animal and cell models, provide critical information for defining therapeutic concepts. Broad tumour cell panels have been used for many years to gain insight into the breadth of potential effects of a given molecule, compare similar drug mechanisms and derive patient stratification biomarkers. More recently, with the expansion of available tumour patient-derived xenograft (PDX) models, the same approach has been taken to assess the likely breadth of response in multiple *in vivo* models. These large datasets help build confidence and refine choices in drug development but, more importantly, the negative data in these experiments enable full contextualisation of the opportunities and risks with a target or therapeutic approach.

The ability to generate increasingly complex disease models – for instance, those that incorporate diverse genetics or intact immune systems – has given rise to many high-profile publications (for example, Sánchez Rivera and Dow, 2024; Lannagan et al., 2021) that introduce new targets or therapeutic concepts with significant implications for treating human disease. However, these models are challenging to develop, and expensive to breed and characterise in order to generate the large amount of data required by the complexity of the systems, meaning that these studies commonly use only a single model. Drawing parallels with what we have learnt from using cell and PDX tumour panels, work in other models is crucial to broaden our understanding of relevant concepts. Taking this further, great insights can be derived from experiments in which unexpected results are observed, or from studies carried out in the same models but in different laboratories or animal facilities. The number of data points obtained from individual experiments can be quite limited, so the availability of data from multiple experiments can be of great use to quickly position a new concept and guide a more robust decision-making process. This makes access to all data, positive or negative, from all studies on multiple models hugely beneficial for any future experimental design and for the interpretation of results (Calza et al., 2021; Editorial, 2023; Errington, 2024; Gamo et al., 2017; Seyhan, 2019; Truebel and Thurston, 2020).

## Emerging initiatives and trends

### Open publishing and data sharing

Given the development of preprint servers such as SSRN, bioRxiv and medRxiv, it was predicted that more negative data would be shared with the community. However, generating a full publication based on negative or unexpected results is still a rare occurrence in the biomedical field and it tends to be limited to high-profile rebuttals (Bassetto et al., 2023; May et al., 2023). Facilitated ways of rapid publishing, such as the Wellcome Open Resource (https://wellcomeopenresearch.org/), F1000 Research (https://f1000research.com/) or other micropublication resources (https://www.micropublication.org/), which accept smaller articles or single experiments, are a big step in the right direction. These initiatives allow smaller or individual results to be published that would not warrant full publication. Of course, these studies still need to be robust but can act as an early disseminator for the community.

However, the general consensus remains that publication of such negative studies is still a long endeavour that requires multiple experiments, especially when aimed at refuting an original study, as well as a rigorous review process. In the mouse genetics field, for example, many claims of lineage specificity by Cre recombinase-expressing lines have been refuted by subsequent studies, whereas multiple studies initiated by laboratories in the meantime could have been prevented if rapid publication of caveats was possible.

### Essential metadata

Although not strictly described as negative data, missing metadata have a similar impact.

Guidelines such as Animal Research: Reporting of *In Vivo* Experiments (ARRIVE) (du Sert et al., 2020) decree precise reporting of animal experiments in peer-reviewed publications, but these are often incomplete or incorrect and overlooked in the editorial process. Likewise, when examining data deposited in archives, such as the Sequence Read Archive (SRA; https://www.ncbi.nlm.nih.gov/sra), key pieces of metadata are often missing and, when present, different terms can be used to describe identical features in different studies. This information can be as crucial as the sex of the animal (Justice, 2024) or, for example, in the cancer field, the origin of the analysed tumour (primary or metastasis). Moreover, the addition of complete and correct metadata would highlight potential causes of non-reproducibility, spur investigations into the biological causes of variation between studies and encourage the publication of contradictory data examining these effects.

A crucial element for reproducibility is to establish a consensus on the key metadata that are required to describe the experiment, along with the specific terminology used to capture these data consistently and the workflows used to preprocess the experimental data prior to analysis. Initiatives such as the Medical Research Council (MRC) National Mouse Genetics Network in the UK (https://nmgn.mrc.ukri.org/) require common metadata to be assembled from all member laboratories (over 100 of which work within the Network). This reflects a recognition of the importance of assembling datasets that can be shared according to ‘Findable, Accessible, Interoperable and Reproducible’ (FAIR) principles. Moreover, the ability of the Network to measure larger sets of parameters that might elucidate some of the variation observed between studies as well as between animal facilities may yield further insights into differences and highlight the difficulty of reproducing certain datasets.

### Sharing preclinical experiments at an early stage

To encourage all data generated using animal studies to be publicly available at some point, an understanding of the data that is being collected is required. Pre-registration of single studies (https://www.animalstudyregistry.org or https://preclinicaltrials.eu/) or reporting of the study design prior to data collection using guidelines such as those of the Open Science Framework (https://osf.io/) is increasing in popularity. Even though these initiatives are supported by an increasing number of journals and organisations (https://www.cos.io/about), the number of studies in which hypotheses, experimental designs and details on statistical analysis are explicitly published prior to the start of experimental work represents a tiny proportion of the overall number of animal studies (Van Der Naald et al., 2022).

PreclinicalTrials EU (https://preclinicaltrials.eu/) enables registration of any kind of preclinical animal study from any country or organisation with the option of comparing the end results with the planned experiment. They offer an optional 5-year embargo, although only a quarter of the almost 200 trials are under embargo, highlighting the community acceptance of the data-sharing model promoted by the platform to avoid duplications and to encourage transparency.

FC3R Short Notes (https://www.fc3r.com/en/FC3R-short-notes.php) proposes the publication of data based on an assessment of quality criteria, such as writing, figures and experimental design, as opposed to novelty and impact. This potentiates a rapid process leading to digital object identifier (DOI) attribution and a dedicated page on the FC3R website. This approach could be adopted more generally by other organisations and journals, reducing the huge volume of unpublished results that languish in laboratory books and computers around the world, ultimately encouraging better-informed research project design in the future.

Cancer Research UK is working in concert with a number of journals (e.g. PLOS) to set up a pilot of pre-registration of animal experiments (https://www.cancerresearchuk.org/funding-for-researchers/how-we-deliver-research/positive-research-culture/registered-reports). These are reviewed prior to commencement of the study to support best practice and the assessment of the importance of the research question. These reviews also ensure that grants that are submitted to Cancer Research UK have their animal components very carefully scrutinised. After the completion of the study, this should allow the study to be more publishable, whatever the outcome, given the agreement by the journal over the importance of the question.

A key consideration, however, is how these studies will be assessed by the community in an era of ever-increasing publication, deposition of data and overload of information. In this context, the dissemination and annotated promotion of the available resources will play a pivotal role in guaranteeing the success of each initiative and will need the support of publishers (Box 1) and technology platforms.

**Box 1. How Disease Models & Mechanisms supports data dissemination**

Disease Models & Mechanisms (DMM) publishes research in disease biology that has significant translational impact at the interface of basic and clinical science. The journal recognises that negative data are generated from robust and thorough experimentation, and that they can advance our understanding of the mechanism of disease, as well as impact its diagnosis and treatment. In these situations, DMM encourages authors to include these negative data in their manuscript. However, if quality negative data do not significantly advance our understanding of human disease, DMM's publisher, The Company of Biologists, still endeavours to support this research by offering a ‘One-Click’ transfer to Biology Open (BiO), which considers rigorous reports presenting negative results.

DMM also encourages transparency and reproducibility in research. The journal requires that authors follow best practice in data reporting, in part, by following the Animal Research: Reporting of *In Vivo* Experiments (ARRIVE) guidelines and depositing primary data for high-throughput experiments in an appropriate public database. DMM also does not set word limits for the Materials and Methods section, to encourage reproducibility and fully informed interpretation of results. The Company of Biologists continues to evaluate and improve its publishing practices to ensure that it is fully supporting our communities.

## Conclusions

In conclusion, preclinical research would be considerably further forward if the dissemination of negative results with accompanying metadata was standardised. It is not surprising that in a profession in which we seek conclusive, ground-breaking and scientifically novel results, researchers are not enthused by the prospect of writing up disappointing results. However, it is clear that the way forward is by facilitating the ease of publication and by increasing recognition for these efforts by peers and funders. We are a long way from the day when all valid data generated in animal facilities and laboratories are freely accessible, but we have an ethical and moral duty to pursue this end.
